# Perceptions, practices and barriers to reporting of adverse drug reactions among HIV infected patients and their doctors in 3 public sector hospitals of the Ethekwini Metropolitan, Kwa-Zulu Natal: a cross sectional and retrospective analysis

**DOI:** 10.1186/s12913-022-08395-3

**Published:** 2022-08-18

**Authors:** Sindiswa Zondi, Panjasaram Naidoo

**Affiliations:** Discipline of Pharmaceutical Sciences, School of Health Sciences, University of Kwa-Zulu Natal, P.O. Box X5401, Durban, 4000 South Africa

**Keywords:** Adverse Drug Reactions, HIV, Doctors, Pharmacovigilance, Perceptions, Practices, PLWHA

## Abstract

**Background:**

Adverse drug reactions (ADRs) remain a global public health concern. Pharmacovigilance practises are essential in ensuring patients safety and post drug marketing surveillance. This study aimed to describe practices, perceptions and barriers towards ADR reporting practices amongst People Living with HIV/AIDS (PLWHA), who are on Highly Active Anti-Retroviral Therapy (HAART) and their doctors.

**Methods:**

The study took place at 3 public sector hospitals. The first phase of the study was a quantitative cross-sectional study using a closed ended questionnaire that was given to PLWHA. Phase two was a retrospective analysis of these patients’ medical files, whilst phase 3 included a descriptive statistics to determine the frequencies and percentages for variables such as ADR reporting practices by doctors**.**

**Results:**

Spontaneous reporting, was evident with 202 patients (48%) indicating that they reported experiencing ADRs to their doctors. Ten doctors (77%) indicated that they received PV training. Eight (62%) doctors indicated that the completed ADR reporting forms were submitted to the pharmacy manager in the hospital for forwarding to the regulatory authority, with 2 (15%) indicating that they submitted directly to the South African Health Products Regulatory Authority. Four (31%) doctors stated that the system of reporting ADRs is ineffective with the majority of the doctors 12 (92%) responding that the reporting of ADRs is very important/critical. A barrier cited by 4 patients (0.9%) for non-reporting of their ADRs was transport cost. Whilst doctors' barriers included reporting being time consuming (31%), and a lack of availability of reporting forms (31%).

**Conclusion:**

Patients and doctors are reporting ADRs but more education and easier reporting process should be available to strengthen the knowledge and reporting of ADRs. Doctors agree that it is critical to report ADRs. Electronic reporting should be encouraged to lessen the time it takes to report ADRs.

## Background

The 2020 Joint United Nations Programme on Human Immunodeficiency Virus/Acquired Immunodeficiency Syndrome (UNAIDS) report states that there are 37.7 million people living with Human Immunodeficiency Virus (HIV) globally [1]. The Eastern and Southern African region has the highest number of people living with HIV i.e. 20.7 million [1]. Furthermore, there are 7.5 million people living with HIV and Acquired Immunodeficiency Syndrome (AIDS) in South Africa [2]. Despite the efficacy of Highly Active Anti-Retroviral therapy (HAART) to treat HIV disease, like all other medicines, it is also associated with risk and in the case of HAART, findings indicate that it is also associated with Adverse Drug Reactions (ADRs) [3, 4]. ADRs are a cause of mortality and morbidity globally [5]. World Health Organization (WHO) defines ADRs as “any response to a drug which is noxious and unintended, and occurs at doses normally used in man for prophylaxis, diagnosis or therapy of disease, or for the modification of physiologic function” [5].

The detection of rare ADRs is often unachievable in clinical trials and some ADRs only present post marketing, this is due to the presence of co-morbidities, lack of long-term use, concomitant use of medicines, minimal number of patient exposure and diversity in the patient population [6, 7]. For this reason, Health Care Professionals (HCPs) and patients ought to report ADRs detected post marketing. WHO defines Pharmacovigilance (PV) as “the science and activities relating to the detection, assessment, understanding and prevention of adverse effects or any other drug related problems” [5]. One of the challenges experienced by HCP is feedback. This was confirmed in a South African study where a pharmacist was reported to say “you report ADRs in a vacuum, you give it to somebody and you never hear again and it’s nice to get feedback, from whoever is collecting these ADRs to say, look, this is what we’re looking for, this is not what we’re looking for” [8].

The three main key role players in the South African PV system are HCPs, pharmaceutical industry, and the public [9]. The South African Health Products Regulatory Authority (SAHPRA) is the medicines regulatory body of South Africa [3]. SAHPRA has a pharmacovigilance unit that monitors ADRs known as the National Adverse Drug Event Monitoring Centre (NADEMC). NADEMC collates and manages ADRs voluntarily reported by HCPs [3]. The reported ADRs are then relayed from NADEMC to SAHPRA [10]. Data collected by the national ADR database in South Africa is further relayed to the Uppsala Monitoring Centre’s VigiBase [11]. South Africa has collected only 28,609 ADRs which were reported to VigiBase since it joined WHO International Drug Monitoring Programme in 1992 [12]. This indicates low reporting as it was found that there have been 27 ADR reporting forms per million per year collected [13]. Some of the barriers to the proper management of data relayed to SAHPRA is underreporting, poor data base compatibility, malfunction of the electronic system, no public relations officers and HCPs not receiving feedback [10, 14].

Spontaneous reporting which is also called passive surveillance, targeted spontaneous reporting and cohort event monitoring are PV surveillance methods that have been implemented and are practiced in South Africa [15]. Spontaneous reporting is defined as “an unsolicited communication by health care professionals or consumers that describes one or more adverse drug reactions in a patient who was given one or more medicinal products and that does not derive from a study or any organized data collection scheme” [16]. Spontaneous reporting has been identified as an easy and an ideal form of reporting ADRs used by HCPs in many PV systems [5, 9, 17]. The spontaneous reporting of ADRs by HCPs is low and it is not mandatory in most countries [18, 19].

Cohort event monitoring is an active surveillance tool that is used to monitor and document ADRs experienced by patients who have been enrolled to investigate the effects of the prescribed medication, furthermore in this method, there is no interference despite the severity of an ADR [20]. Targeted spontaneous reporting is a novel PV tool that conjoins elements of spontaneous reporting and cohort event monitoring [21]. There is an identification of a specific group of patients who have been described a specific drug or regimen and the subsequent ADRs are monitored in a cohort setting [21].

Enhancing drug safety to alleviate patient harm ought to be a shared responsibility of amongst key holders. Drug safety practices should not be limited to new medicines but rather all medicines should be monitored and reported for associated ADRs in order for new knowledge to be constantly generated regarding disease and medicine [5]. The responsibility of the ongoing collection of ADRs associated with medicines mainly lies with the pharmaceutical industry however post marketing, consumers play an important part. They are most likely to report ADRs to their HCPs compared to communicating them directly to the pharmaceutical industry. Therefore HCPs play a critical role in reporting ADRs to ensure a proper PV system is available and functional [22, 23]. The aim of this study was to gauge and describe practices, perceptions and barriers towards the reporting of ADRs by PLWHA on HAART and their doctors.

## Methodology

### Study site

The study was carried out at 3 public sector hospitals situated in the eThekwini Metro area of Kwa-Zulu Natal, South Africa. These hospitals are all similar in size and are categorized as regional hospitals by the South African Health Care System. The hospitals had to have an antiretroviral (ARV) outpatient clinic, attached to it in order to qualify as a research site. They were coded as Facility A, B and C. Facility A and B are in residential areas and can be accessed by residents through walking. Facility C is in a tourist/commercial area that is accessible by means of transportation. All three facilities have qualified nurses who do administration. Nurses also take vitals such as weight and draw blood for biochemical assays. The clinics also have doctors who manage patients and prescribe ARVs. The facilities also have pharmacists who dispense ARVs and counsel patients on the proper use of their medication as well as give information to doctors and other health care providers on medications.

### Study design and instrument

The study was divided into three phases. The first phase was the quantitative cross sectional study which entailed the distribution of close ended questionnaires to patients on HAART. The second phase was a retrospective analysis of these patients' medical files. The purpose of the retrospective analysis was to describe the ADRs associated with HAART experienced by PLWHA. Another purpose of the retrospective analysis was to correlate and confirm if the ADRs stated on the questionnaire were really experienced by patients. Therefore the medical files were transcribed to describe experienced ADRs from the period 1991 to 2019. ADRs are unexpected therefore retrospective analysis was appropriate to document past experienced ADRS. Phase 3 of the study included descriptive statistics to determine the frequencies and percentages for variables such as ADR reporting practices by doctors, education of patients by doctors and barriers to reporting. The closed and open ended questionnaires were distributed to doctors who manage these cohort of patients described in phase 1. The cross sectional study took place between the 2^nd^ of December 2019 and the 31^st^ of January 2020, whilst the retrospective study analysis dated back from 1991–2019. The questionnaire was developed by looking at questions from similar studies, looking at other questionnaires and mostly from an in depth literature review [24–29]. The questions were constructed to achieve information on the objectives of the study.

The pretesting of the questionnaire was done by giving the questionnaire to 3 doctors in the facilities where the study was carried out, but these doctors were not involved in the ARV clinic, hence were not part of the sample population. A total of 3 doctors participated in the pilot study and gave their input. With regards to the patient questionnaire post graduate students at the university were invited to participate in the pilot study and give their input. Suggestions from the participants in the pilot study were adopted, therefore the questionnaires were further amended to exclude race as a variable.

### Study population

All PLWHA who attended these selected sites and their respective doctors who managed them formed the study population. PLWHA who had been on HAART for more than 6 months and were 18 years and older during the study period were included. All HCPs who managed these patients were included in the study. The study only targeted doctors who managed outpatient HIV/AIDS patients. HIV patients younger than 18 years and those less than 6 months on HAART were excluded.

### Sampling techniques and calculation of sample size

The total target population of patients using HAART in the Metro was 383,869 during the time of the study [30]. Sample size was calculated using single population proportion formula ${\varvec{n}}=\frac{\frac{{{\varvec{Z}}}^{2}{\varvec{P}}(1-{\varvec{P}})}{{{\varvec{d}}}^{2}}}{1+\frac{{{\varvec{Z}}}^{2}{\varvec{P}}(1-{\varvec{P}})}{{{\varvec{N}}{\varvec{d}}}^{2}}}$, using 95% confidence level, 5% degree of precision, 50% of expected number of patients with the number of ADRs [31]. The sample size was calculated to be 384, the possible dropout was considered, and therefore 10% was added to make the sample size 423.

The sample size was distributed amongst three ARV facilities hence the sample size for each hospital was 141.

Prior to commencing the study, permission was sought from the hospitals and ethical approval was obtained from the Institution. All the HCPs who prescribe HAART in the three facilities were asked to participate in the study. The ethics approval number being BE053/19.

### Data collection procedure

In each study facility, about 400 patients came to the clinic daily. During the study period, patients awaiting consultation with a doctor were approached as a group and briefed on the study to obtain their consent to participate in the study. The informed consent, anonymity, voluntary participation, inclusion criteria were communicated with the patients. Patients who consented to enroll in the study were given a coded closed ended, anonymous questionnaire to fill. The purpose of the coding was to match the questionnaire to the patient’s clinical file which was the second phase of the study. The questionnaire was available in both English and IsiZulu, the latter being the most common language spoken in the eThekwini Metro and South Africa generally [32]. The questionnaire required information on their demographics, confirmation of ADRs experienced and the reporting of ADRs. The questionnaires were collected immediately after completion. The researcher ascertained that the questionnaires are returned after completed by encouraging the participants to return the questionnaires. This was done by informing the participants that they can continue with the questionnaire even after the consultation with doctor whilst waiting for medicine to be dispensed by the pharmacist, if the completion was of the questionnaire was disturbed by the doctor’s consultation. However it is not all the approached patients who returned the questionnaires. All the data collected was treated confidentially.

The HCPs were approached, the study explained, and consent obtained before the coded questionnaire was given to them. The HCPs were given a week to complete the questionnaires.

### Data analysis

All analysis was conducted using SPSS version 25. Continuous and categorical variables such as patients and doctors demographics, years of experience post intern, and the number of ADRs reported were compared using the descriptive analysis to determine frequencies and percentages.

## Results

### Response rate

#### Socio-demographic information of patient participants

Table 1 describes the socio-demographic information of the PLWHA participants. Of the 426 respondents, 296 (69%) respondents were females, 126 (30%) were males and 4 (1%) were transgender respondents. Two hundred and three (52%) patients had secondary school education, whilst 113 (27%) had primary school level of education, 71 (17%) having tertiary education and 12 (3%) patients having no educational background. The respondents ranged from 18 to 69, with the median age of 41 years (IQR 39.5 to 41.7). Two hundred and twenty (52%) patients were unemployed, 126 (30%) were employed with 61 (14%) being patients that were self-employed while 16 (4%) were receiving a pension grant. Three hundred and fifteen (74%) patients travelled by taxi, 49 (12%) walked, 18 (10%) travelled by bus, 37 (9%) used own car and 4 (0.9%) travelled by train. Two hundred and forty four (57%) patients lived in the township, 82 (19%) urban area, 70 (16%) rural area, 25 (6%) semi-urban.

**Table 1:** Socio-demographic information of patient participants

Variable	Facility A (*n* = 141)		Facility B (*n* = 141)		Facility C (*n* = 144)	
	Frequency	Percentage	Frequency	Percentage	Frequency	Percentage
**Number of patient participants**						
Number of patients (*n* = 426)	141	33%	141	33%	144	34%
**Age**						
18– 35 years	44	31%	60	42%	47	32%
36– 55 years	80	57%	69	49%	76	53%
56 + years	17	12%	12	9%	21	15%
Median age was 41 years (IQR 39.5 to 41.7)						
**Gender**						
Female	104	74%	95	67%	97	67%
Male	37	26%	46	33%	43	30%
Transgender	-	-	-	-	4	3%
In all 3 facilities the majority were females. Overall: females 296 (69%), males 126 (30%), transgender 4 (1%)						
**Educational level**						
Primary Education	51	36%	44	31%	18	13%
Matric	72	51%	64	45%	87	60%
Tertiary	13	9%	19	13%	39	27%
No schooling	4	3%	8	6%	-	-
Overall: 223 (52%) patients had secondary school education, whilst 113 (27%) had primary school level of education, and 71 (17%) having tertiary education with 12 (3%) patients having no educational background						
**Employment**						
Self-employed	24	17%	23	16%	14	10%
Employed	42	30%	38	27%	46	32%
Non-employed	69	49%	77	55%	74	51%
Pensioner	6	4%	1	1%	9	6%
Overall: 220 (52%) patients were unemployed, 126 (30%) were employed with 61 (14%) being patients that were self-employed while 16 (4%) were receiving a pension grant						
**Mode of transportation used when coming to the facility**						
Walk	12	9%	19	13%	18	13%
Bus	5	4%	3	2%	10	7%
Taxi	113	80%	104	74%	98	68%
Train	1	0.7%	1	0.7%	2	1%
Own car	10	7%	11	8%	16	11%
Overall: 315 (74%) travelled by taxi, 49 (12%) walked, 18 (10%) travelled by bus, 37 (9%) used own car, 4 (0.9%) travelled by train						
**Residential area**						
Urban area	19	4%	8	2%	55	13%
Rural area	24	6%	32	8%	14	3%
Township area	92	22%	90	22%	62	15%
Semi-urban area	6	1%	7	2%	12	3%
Overall: 244 (57%) lived in the township, 82 (19%) urban area, 70 (16%) rural area, 25 (6%) semi-urban						

#### ADRs’ reporting practices amongst patient participants

In facility B, 4 patients indicated that they lacked funds as a barrier to reporting an ADR (Table 2). Two of whom matriculated, whilst one had primary school education and 1 had no educational background. Three of these patients were unemployed whilst the one participant who was employed was a matriculant. All four patients resided in a township and had to use a taxi to travel to the facility.

**Table 2:** Self reporting ADRs’ practices amongst patient participants

Question/ variable	Facility A (*n* = 141)		Facility B (*n* = 141)		Facility C (*n* = 144)	
	Frequency	Percentage	Frequency	Percentage	Frequency	Percentage
Number of patients who experienced an ADR	86	61%	117	83%	103	72%
**Did you report your ADR at the clinic?**						
Yes	54	38%	82	58%	66	45%
No	2	1%	19	13%	6	4%
**When did you report an ADR?**						
Within a week	2	1%	20	14%	-	-
Less than a month	1	0.7%	27	19%	-	-
After a month	47	33%	31	21%	66	46%
**What led to no reporting?**						
No money to go to the clinic	-	-	4	3%	-	-
Did not find it necessary to report at the clinic	-	-	1	0.7%	2	1%
Consulted a traditional healer	-	-	3	2%	-	-
**Did the HCP inform you that you might experience an ADR?**						
Yes	114	80%	109	77%	107	74%
No	15	10%	29	20%	30	21%

In facility B, non-reporting by 3 patients was due to them consulting a traditional healer. Of these 3 patients, two resided in a township whilst one resided in a rural area.

One patient from Facility B and 2 patients from Facility C did not report an ADR as they found it was not necessary to do so even though one patient from Facility B and 1 from Facility C were informed by their doctor of possible ADRs and to report.

#### ADR reporting practices amongst patient participants hospitalized due to ADRs

Table 3 describes the reporting practices of the 36 patient participants who were hospitalised due to experiencing an ADR.

**Table 3:** ADR reporting practices among hospitalized patient participants (*n* = 36)

Question/ variable	Facility A (*n* = 7)		Facility B (*n* = 22)		Facility C (*n* = 7)	
	Frequency	Percentage	Frequency	Percentage	Frequency	Percentage
**Did you report your ADR at the clinic?**						
Yes	4	57%	14	63%	7	100%
No	3	42%	5	22%	-	
**When did you report an ADR?**						
Within a week	-	-	5	22%	-	
Less than a month	-	-	2	9%	-	
After a month	4	57%	6	27%	7	100%
**What led to no reporting?**						
No money to go to the clinic	-	-	2	9%	-	
Consulted a traditional healer	-	-	2	9%	-	
**Did the HCP inform you that you might experience an ADR?**						
Yes	5	71%	17	77%	5	71%
No	1	14%	5	22%	2	29%

Two hospitalized patients that did not report an ADR due to transport costs whilst the other 2 consulted a traditional healer.

Mortality rate was zero for the 36 patients who were hospitalized due to ADRs.

#### Retrospective analysis of patient participants’ medical files yielded the following results

Three hundred six (72%) of patients experienced an ADR as recorded on their files.

#### Demographic characteristics of doctor participants

The response rate was 100%. HCPs who responded were 13 in number and were all medical doctors that managed the patient participants.

The demographics of the doctor participants are described in Table 4.

**Table 4:** Demographics of the doctor participants

Demographic characteristics	Facility A (*n* = 6)		Facility B (*n* = 4)		Facility C (*n* = 3)	
	Frequency (n)	Percentage (%)	Frequency (n)	Percentage (%)	Frequency (n)	Percentage (%)
**Gender**						
Male	4	67%	2	50%	1	33%
Female	2	33%	2	50%	2	67%
**Number of years working post intern**						
1–10 years	2	33%	1	25%	1	33%
11–20 years	3	50%	1	25%	2	67%
21–25 years	-		1	25%	-	-
> 25 years	1	17%	1	25%	-	-
**Number of years working with HIV patients**						
0–5 years	1	16%	-	-	1	33%
5–10 years	4	67%	2	50%	2	67%
10–15 years	1	16%	3	50%	-	-

In Facility C there was no doctor who had more than 10 years’ experience of working with HIV infected patients.

#### Training of doctor participants on pharmacovigilance

In Facility A, all 6 doctors indicated that they received PV training in their undergraduate studies.

In Facility B, there were 3 doctors who indicated that they received PV training in their undergraduate study, of these 3 doctors, 1 doctor did a postgraduate supplementary course on HIV and PV.

In Facility C, there was 1 doctor who received PV training in the undergraduate years, whilst 2 doctors did postgraduate supplementary training.

##### Open ended responses by doctor participants on training:

- 1. Have more workshops educating HCP on PV.
- 2. Have seminars for intern doctors regarding ADRs and PV.

##### ADR diagnosis and form submission practice of the doctor participants and education of patients by doctors

In facility A, 2 (33%) doctors did not indicate where they submit completed ADR forms, of these two doctors one indicated that he or she never fills in an ADR form and another indicated to sometimes completing an ADR form (Table 5).

**Table 5:** ADR diagnosis and form submission practice of the doctor participants and education of patients by doctors

Question/variable	Facility A (*n* = 6)		Facility B (*n* = 4)		Facility C (*n* = 3)	
	Frequency	Percentage	Frequency	Percentage	Frequency	Percentage
**How do you diagnose ADRs?**						
Laboratory results	5	83%	4	100%	3	100%
Patient clinical examination	6	100%	4	100%	3	100%
Ask patient questions	6	100%	4	100%	3	100%
Using WHO definition of an ADR	4	67%	3	75%	1	33%
South African ART Guidelines	5	83%	4	100%	3	100%
Consulting medicine/ surgery/ medical science textbook and or journals	3	50%	3	75%	1	33%
All doctors diagnose ADR by questioning the patient and examining them with doctors in Facility B and C using laboratory results and the SA ART guidelines as well						
**Where must a completed ADR form be submitted?**						
Pharmacy manager	2	33%	3	75%	3	100%
SAHPRA	2	33%	-	-	-	-
**How often do you fill in the ADR form?**						
Never	3	50%	-	-	-	-
Sometimes	1	17%	1	25%	-	-
Often	-	-	-	-	1	33%
Always	1	17%	3	75%	2	67%
**In an event of serious (life threatening/causes birth defect/requires hospitalization) ADRS, when do you report it?**						
1 day	2	33%	4	100%	2	67%
**In an event of non-serious ADR, when do you report it?**						
1 day	2	33%	4	100%	2	67%
1 week	-	-	-	-	1	33%
**In an event where by you prescribed a newly produced ARV drug and there is an unexpected ADR which can help contribute towards new information relating to benefit-risk profile of the new drug, when do you report it?**						
1 day	1	17%	4	100%	1	33%
2 days	4	67%	-	-	-	
1 month	-	-	-	-	1	33%
**Is there Wi-Fi in your hospital in order to carry out fast, efficient and easy reporting of ADRs if online system is implemented?**						
Yes	-	-	-	-	3	100%
No	6	100%	4	100%	-	-
Summary of open ended responses: 1. Lack of feedback/report back is a concern 2. Perception that spontaneous reporting is used as a marketing tool						
**How often do you educate patients about ADRS?**						
Sometimes	1	-	-	-	-	
Often	1	17%	-	-	2	67%
Always	4	67%	4	100%	1	33%

The doctor participant in facility A, who does not use laboratory results to diagnose ADRs, had less than 10 years’ experience post intern and working with HIV patients, furthermore the same doctor did not respond to where the ADR form ought to be submitted. The same doctor indicated that it is critical to report an ADR however indicated to never completing an ADR form when patients experience an ADR (Table 5).

There were 9 (69%) doctor participants who responded that they always educate patients about ADRs (Table 5).

Only 8 (62%) doctors indicated they report an ADR whether it is serious or not serious with in a day. However if it is a new drug, 10 doctors indicated that they would report the ADR within a day or 2 days, however 1 doctor indicated to reporting ADRs associated with new medicines in a month (Table 5).

There were 330 (77%) patients who were warned by doctors that they might experience an ADR. There were 306 (72%) patients who experienced an ADR, 202 (47%) of which reported experienced ADRs at their facilities. There were 104 (24%) patients who did not report experienced ADRs despite having 9 (69%) doctors in the 3 facilities indicating that they always educate patients regarding ADRs.

### Perceptions towards ADR practices among doctor participants

All doctors stated that electronic spontaneous reporting of ADRs would be more effective than the manual submission of ADRs, and that SAHPRA/Department of Health/NADEMC’ should host more pharmacovigilance workshops as they felt it would improve the practice of pharmacovigilance and reporting;

They also perceived PV procedures as being essential for medicine safety.

There were 6 (46%) doctors who responded that it is critical to carry out spontaneous reporting of ADRs. The 1 doctor who indicated that it was difficult to fill in an ADR reporting form was a 10 to 15 years post intern doctor and had between 5 to 10 years’ experience of working with HIV infected patients. This doctor also did not have PV supplementary training.

### Barriers to reporting ADRs and doctor participants

Doctors were asked to indicate barriers that limit their ADR reporting practices. Table 8 describes the barriers as stated by the doctor participants in the different facilities.

The 2 doctors who did not find time as a barrier had over 21 years’ experience as doctors. The doctor in facility A, who indicated to being afraid of taking responsibility, did not respond as to whether the ADR form is completed or not. The doctor in facility C who also responded to being afraid of taking responsibility in reporting ADRs also responded that the current PV system is inefficient.

## Discussion

This study gave a fair overview on the reporting practices of both PLWHA and their doctors. In addition, it also gave some information on doctor’s perceptions and barriers experienced by doctors in the reporting of ADRs.

It is evident that even though patients had been informed by their doctors to report an ADR, they did not do so (Table 6) (77% were warned that they might experience an ADR versus 47% reported ADRs at their facilities). They cited various reasons such as lack of funds to go back to the clinic, or used alternative healers such as traditional healers to manage their ADR or did not find it necessary to do so (Table 2). These findings were similar to a study that was conducted in Nigeria whereby 89 (24.7%) patient participants experienced at least one ADR and 38 (39%) reported experienced ADRs to HCPs [33]. In a study that was conducted in Malaysia there were 240 (72%) patient participants that were informed by doctors of the possibility of experiencing ADRs, 233 (66.8%) reported experienced ADRs to the doctors [34]. Furthermore, in this study, where no self-reporting of an experienced ADR was stated, it was found that ADRs were recorded on the patients' files. This could possibly be due to the patients not being able to differentiate between an ADR and an Adverse Drug Effect (ADE) or the seriousness of the former. Poor knowledge could have led to this behaviour.

**Table 6:** Summary of ADR reporting practises among patient and doctor participants

Question/variable	Facility A		Facility B		Facility C	
	Patients (*n* = 141)		Patients (*n* = 141)		Patients (*n* = 144)	
	Doctors (*n* = 6)		Doctors (*n* = 4)		Doctors (*n* = 3)	
	Frequency	Percentage	Frequency	Percentage	Frequency	Percentage
Number of patients who were warned by doctor that they might experience an ADR	114	80%	109	77%	107	74%
Number of patients who experienced an ADR	86	61%	117	83%	103	72%
Number of patients who reported ADRs	54	38%	82	58%	66	45%
Number of patients who did not report experienced ADRs	32	23%	35	25%	37	26%
Number of doctors who always educate their patients about ADRs	4	67%	4	100%	1	33%

Reasons for some patients not reporting ADRs in our study, included patients in facility B and C, who indicated that they did not find it necessary to report an ADR at their facilities, this might be due to these patients not deeming the reporting of ADRs as important and might have concluded that the experienced ADRs as non-serious. Another reason cited by patients for not reporting was the management of ADRs by traditional healers. The consultation of traditional healers to treat ADRs that were caused by western medication is a concern and it can be assumed that these patients sought treatment from a traditional healer because these traditional healers were easily accessible and lived nearby to these patients. Of these 3 patients, 2 were eventually hospitalized. In addition, 8% of the patients in our study were hospitalized due to ADRs (Table 3). The timeous reporting of ADRs could lead to ADRs’ preventability and reduction in the financial burden and hospital costs associated with ADRs. As cited in studies, 1 in 10 adult admissions are due to ADRs [35]. South Africa in multi-ethnic base country and the use of traditional medicines is common, furthermore an Ethekwini based study showed that its participants used HAART concurrently with traditional medicines [36]. A study conducted by Abdel-Latif and Abdel-Wahab indicated that in a multi-ethnic country it is essential for HCPs to monitor and report ADRs [37]. The lack of funds cited by some participants who experienced an ADR but did not go to the clinic for management poses a serious threat to the achievement of positive health outcomes. All patients who indicated, that they did not have money to go to the clinic, were from low income areas, with 75% of them being unemployed (Table 1). However of concern is that the location of these hospitals are not remotely situated, are within residential suburbs and can be accessed by foot. It can therefore be assumed that these patients preferred to use transportation to go to the clinic rather than to walk to the clinic if funding was an issue. This however leads to further detriment of their health and increased health costs as ADR is a “response to a drug which is noxious and unintended and which occurs at doses normally used in man for prophylaxis, diagnosis, or therapy of disease or for the modification of physiologic function” and needs urgent attention [38].

Further concern noted in this study was that a third of the patients stated that they reported ADRs after a month with almost 50% of the hospitalized patients indicating that they reported their ADRs after a month. Fortunately there were no deaths from the ADR experienced. This begs the question as to whether patients were informed that they should report an ADR immediately at the clinic. This late reporting is not acceptable as a serious ADR could have led to death. This study further found that the incidence of ADR was high 306 (72%). A finding similar to a study that was conducted by Hagos et al*.,* where by 62.8% patients experienced at least one ADR [39].

Therefore educating patients about ADRs and the importance of reporting immediately and its subsequent management is vital. In addition, it is essential that patients are informed in layman’s terms for better understanding. Knowing the difference between an ADR and an ADE should form part of the education programme for patients. In order to confirm that patients understood what information was given to them, is to ask patients to repeat the information given to them. One of the doctor participant suggested that there ought to be constant PV workshops and in these workshops it should be highlighted that it is crucial for doctors to continuously educate patients about ADRs and also to educate them about the importance of immediate reporting of ADRs.

With regards to doctors’ training, the doctor participants in this study either received PV training during their undergraduate or underwent PV training through supplementary courses, thus the doctors in this study at a minimum had basic PV knowledge to carry out ADR reporting. Maigetter et al., reported that HCPs ought to receive PV training in order to encourage them to participate in PV activities such as the reporting of ADRs [10]. Even though facility C had more patients and fewer doctors, the doctors were all proactive in educating patients regarding ADRs (Table 6). Facility A had more doctors compared to other facilities and it is concerning that only one doctor responded to sometimes educating patients regarding ADRs. Normally shortage of human resources does not allow for much education of patients, but surprisingly in this facility there were more doctors, education of patients was done sometimes.

The practice of these doctor participants was evidenced by ten doctors of the 13 reporting serious and non-serious ADRs within a day or 2 days which is optimum practice however it was concerning that one doctor in facility C indicated that an ADR from a newly registered medicine would be reported within a month (Table 5). In terms of post marketing surveillance, it is important that all ADRs or undocumented ADEs should be reported immediately to the pharmacovigilance unit and/or the manufacturer. This could be once again related to shortage of staff as facility C had fewer doctors managing more patients than facility A and facility B. Half of the doctors in facility A indicated that they never report an ADR which is concerning as the SAHPRA ADR reporting guideline states that HCPs are encouraged to report ADRs even in cases whereby HCPs are uncertain regarding whether a drug caused an ADR or not [40]. The reporting of ADRs associated with new medicines should be done immediately to SAHPRA as this is a safety issue and depending on the severity of an ADR, it ought to be removed from the market. Information about newly registered medicines’ safety and effectiveness is made available after clinical trials [40]. PV is therefore an important tool in the post marketing surveillance and it is critical after clinical trials as post marketing populations have different genetic predispositions and pharmacokinetics and have comorbidities, use traditional medicines which affects the drugs thereby generating undocumented ADRs [9, 41].

A study conducted by Boguluva et al*.,* indicated that 109 (46.8%) of HCPs did not know where to submit the ADR form. This was similar to the findings in this study whereby 23% doctors (2 in facility A and 1 in facility B) did not indicate where the form ought to be submitted, this might due to doctors not knowing where the ADR form ought to be submitted or not being proactive in PV practices [13]. This is concerning as facility B had more patients who experienced ADRs than the other facilities, therefore all doctors in facility B should have been more proactive in reporting ADRs. In spite of these shortcomings the majority of doctors (77%) in this study were compliant with the reporting of ADRs by either submitting the form to the pharmacy manager or directly to SAHPRA (Table 5). The submission of the ADR forms to the pharmacy manager is due to following the hospital protocol for ADR submissions.

One of the open ended responses received stated “I am more interested in the feedback from the data collected during spontaneous reporting” this statement is similar to the findings from a study conducted by Joubert and Naidoo, where all participants indicated that they would like to receive more communication between them and the PV centres [42]. Studies have shown that HCPs not receiving feedback from PV centres discourages HCPs from completing reporting ADRs in the future [43, 44]. As stated in the South African National Development Plan for HIV, TB and STIs 2017–2022, that there ought to the transparency amongst stakeholders and that all stakeholders ought to have access amongst shared data such as data on community response [45].

In this study doctors perceived the reporting of ADR to be either important, very important or very critical practice. This coincides with a South African study which showed that 177 (76%) of the participants indicated that it was very important to report ADRs [13]. A study conducted by Adisa and Omitogun showed that 76 (95%) of their HCPs indicated that the reporting of ADRs as being important [33].

With respect to the PV system, 31% of doctor participants indicated that the system of reporting ADRs is ineffective (Table 7) which was similar to the findings of Joubert and Naidoo where HCPs who participated in the study were lowly satisfied with the PV systems in South Africa. [42]. Some of the barriers cited that affected optimum PV practices in this study included the unavailability of forms and that it was time consuming to report an ADR expressed by almost 50% of the doctor participants (Table 8). In contrast to another study by Khan 2013, which stated that 32% of HCPs agreed to the reporting of ADRs being time consuming with 18% of HCPs strongly agreeing on the unavailability of the ADR reporting forms [46].

**Table 7:** Perceptions towards ADR reporting practices among doctor participants

Question/variable	Facility A (*n* = 6)		Facility B (*n* = 4)		Facility C (*n* = 3)	
	Frequency	Percentage	Frequency	Percentage	Frequency	Percentage
**In an event where by you have diagnosed an ADR from a patient, how important do you think it is to carry out spontaneous reporting?**						
Important	-	-	1	25%	-	-
Very important	2	33%	1	25%	3	100%
Critical	4	67%	2	50%	-	-
**How easy is it to fill the ADR reporting form?**						
Difficult	1	17%	-	-	-	-
Easy	2	33%	3	75%	2	67%
Very easy	-	-	-	-	1	33%
Extremely easy	1	17%	1	25%	-	-
**How efficient is the current system of reporting ADRs?**						
Not efficient	2	33%	1	25%	1	33%
Slightly efficient	2	33%	2	50%	-	-
Moderately efficient	1	17%	1	25%	1	33%
Very efficient	-	-	-	-	1	33%
**Which of the following should be implemented to improve the management of ADRs?**						
Discussing solutions with other HCPs	4	67%	4	100%	1	33%
Education through PV conferences	6	100%	2	50%	3	100%
Increasing supervision of junior doctors to ensure that junior HCPs manage ADRs correctly	6	100%	2	50%	2	67%
Focused research to investigate specific ADRs	3	50%	2	50%	-	-
Open ended responses by doctors:						
1. ADR reporting would be faster if carried out electronically						
2. Anonymous reporting could be better						
3. There should be use of WhatsApp groups

**Table 8:** Barriers to reporting ADRs by doctors (*n* = 13)

	Facility A (*n* = 6)					Facility B (*n* = 4)						Facility C (*n* = 3)				
Variable	Strongly agree n, (%)	Agree n, (%)	Neutral n, (%)	Disagree n, (%)	Strongly Disagree n, (%)	Strongly agree n, (%)	Agree n, (%)	Neutral n, (%)	Disagree n, (%)	Strongly Disagree n, (%)	Strongly agree n, (%)		Agree n, (%)	Neutral n, (%)	Disagree n, (%)	Strongly Disagree n, (%)
Time consuming	3, (50%)	2, (33%)	-	-	-	-	2, (50%)	2, (50%)	-	-	1, (33%)		2, (67%)	-	-	-
Lack of time	2, (33%)	2, (33%)	-	1, (17%)	-	1, (25%)	1, (25%)	2, (50%)	-	-	1, (33%)		-	1, (33%)	-	1, (33%)
Afraid of taking responsibility	-	1, (17%)	-	-	2, (33%)	-	-	2, (50%)	2, (50%)	-	-		1, (33%)	1, (33%)	-	1, (33%)
Forms are not available	3, (50%)	-	2, (33%)	1, (17%)	-	-	2, (50%)	1, (25%)	-	-	1, (33%)		-	1, (33%)	-	1, (33%)
Lacks confidence in discussing the ADRs with other colleagues	-	1, (17%)	-	-	2, (33%)	1, (25%)	2, (50%)	-	-	-	-		2, (67%)	-	-	1, (33%)

All the doctors who participated in this study, indicated, that an electronic reporting of ADRs would be more ideal for the South African PV system to be efficient, the electronic reporting of ADRs would therefore lessen the time it takes to complete a hard copy ADR form and walking to submitting it to the pharmacy manager. It is evident that most African countries utilize the manual system of reporting ADRs [47]. SAHPRA has implemented an electronic reporting of ADRs through an eReporting link which is available at the SAHPRA website [40]. Furthermore SAHPRA has also implemented a MedSafety App which can be downloadable to a cellphone and can be accessed by both HCPs and the public to report ADRs associated with medicines [48]. This therefore suggests that doctors were not knowledgeable with the current electronic reporting tools during the study period as the communications were made in March 2020 and again in April 2021. Even though eReporting has been implemented in South Africa, doctors in Facilities A and B, who indicated they do not have Wi-Fi access may not be able to carry out electronic reporting which will once again pose a barrier to effective and timeous reporting of ADR.

South Africa is a third world country with limited recourses and a burden of HIV/AIDS epidemic. South Africa has one of the largest HAART programmes globally [2]. Continuous research on ADRs associated with HAART is crucial, as ADRs are 'an appreciably harmful or unpleasant reaction, resulting from an intervention related to the use of a medicinal product, which predicts hazard from future administration and warrants prevention or specific treatment, or alteration of the dosage regimen, or withdrawal of the product’ [49]. This makes ADRs a global challenge as they are unpredictable and idiosyncratic and ought to be quantified. This study provides knowledge on the current barriers, perceptions in ADR reporting. Such research is a useful tool in SAHPRA and the Department of Health in order to strategize effective measures to ensure timeous and on-going reporting of ADRs. Reporting of ADRs in pharmaceutical industries is important in order to alleviate risks associated with medicines. However the study carried limitations such as loss of patient files in facilities which posed a barrier in recording experienced ADRs, other limitations included a small sample size of the doctors as the study only targeted doctors who managed outpatient HIV/AIDS patients. The population size of doctors managing outpatient HIV/AIDS patients is smaller compared to the doctors in the main regional hospitals to which the ARV clinics are attached. The non-response bias was among other limitations in this study. It can be assumed that those who did not respond may have had poor knowledge on the objectives of this study. It can also be assumed that when variable under investigation is one that comprises health related variables or is socially undesirable. Some patients did not respond to questions such as whether they experienced diarrhea and depression, as these conditions are socially undesirable. People with risk behaviour are less likely to respond on the questionnaire. The non-response bias affected the study’s generalizability therefore due to the small sample size, the study cannot be generalizable to all PLWHA and doctors who manage PLWHA.

## Conclusion and recommendations

ADRs are associated with HAART, with patients experiencing them at different levels of severity and patienst are aware of ADRs and are report them to doctors; however some patients were still not clear about what to report, ADEs have been incorrectly reported as ADRs in some instances. Doctors have been trained in PV and they report ADRs and the reporting is perceived as being very important to critical. However policies in the hospitals determine where an ADR report should go to, pharmacy managers first before being submitted to SAHPRA. Patients' barriers have also been noted such as lack of travel funds to go to the clinic, whilst the lack of education by doctors resulted in some patients seeking traditional healers to manage their ADR. The doctor barriers that appeared to prevent optimum reporting practices were it being time consuming and more importantly lack of feedback which does not augur well for an efficient PV system. Electronic reporting should be pursued earnestly in order to overcome these barriers.

## Data Availability

All the data that was generated or analyzed during this study is included in this published article.

## Abbreviations

AIDS: Acquired Immunodeficiency Syndrome
ADRs: Adverse Drug Reactions
ADE: Adverse Drug Effects
HCPs: Health Care Professionals
HAART: Highly Active Anti-Retroviral therapy
HIV: Human Immunodeficiency Virus
NADEMC: National Adverse Drug Event Monitoring Centre
PLWHA: People living with HIV/AIDS
PV: Pharmacovigilance
SAHPRA: South African Health Products Regulatory Authority
WHO: World Health Organization
